# First person – Katie Lloyd and Stamatia Papoutsopoulou

**DOI:** 10.1242/dmm.047506

**Published:** 2020-11-27

## Abstract

First Person is a series of interviews with the first authors of a selection of papers published in Disease Models & Mechanisms, helping early-career researchers promote themselves alongside their papers. Katie Lloyd and Stamatia Papoutsopoulou are co-first authors on ‘Using systems medicine to identify a therapeutic agent with potential for repurposing in inflammatory bowel disease’, published in DMM. Katie conducted the research described in this article while a postdoctoral research associate in Prof. Chris Probert's lab at the University of Liverpool, Liverpool, UK. She is now a lecturer in pharmacology at the University of Chester, Chester, UK. Her research focuses on personalising medicine by combining innovative experimental approaches to identify biomarkers of inflammatory disease, drug response and mechanisms of drug resistance, which consider complex factors such as inter-patient variability and co-morbidities. Stamatia conducted the research described in this article while a postdoctoral research associate in Werner Muller's lab at the University of Manchester, Manchester, UK. She is currently a postdoctoral research associate in the lab of Mark Pritchard at the University of Liverpool, Liverpool, UK, investigating the regulation of transcriptional responses during inflammation and the impact of environmental factors on them, and has just accepted the position of assistant professor at the University of Thessaly, Greece.


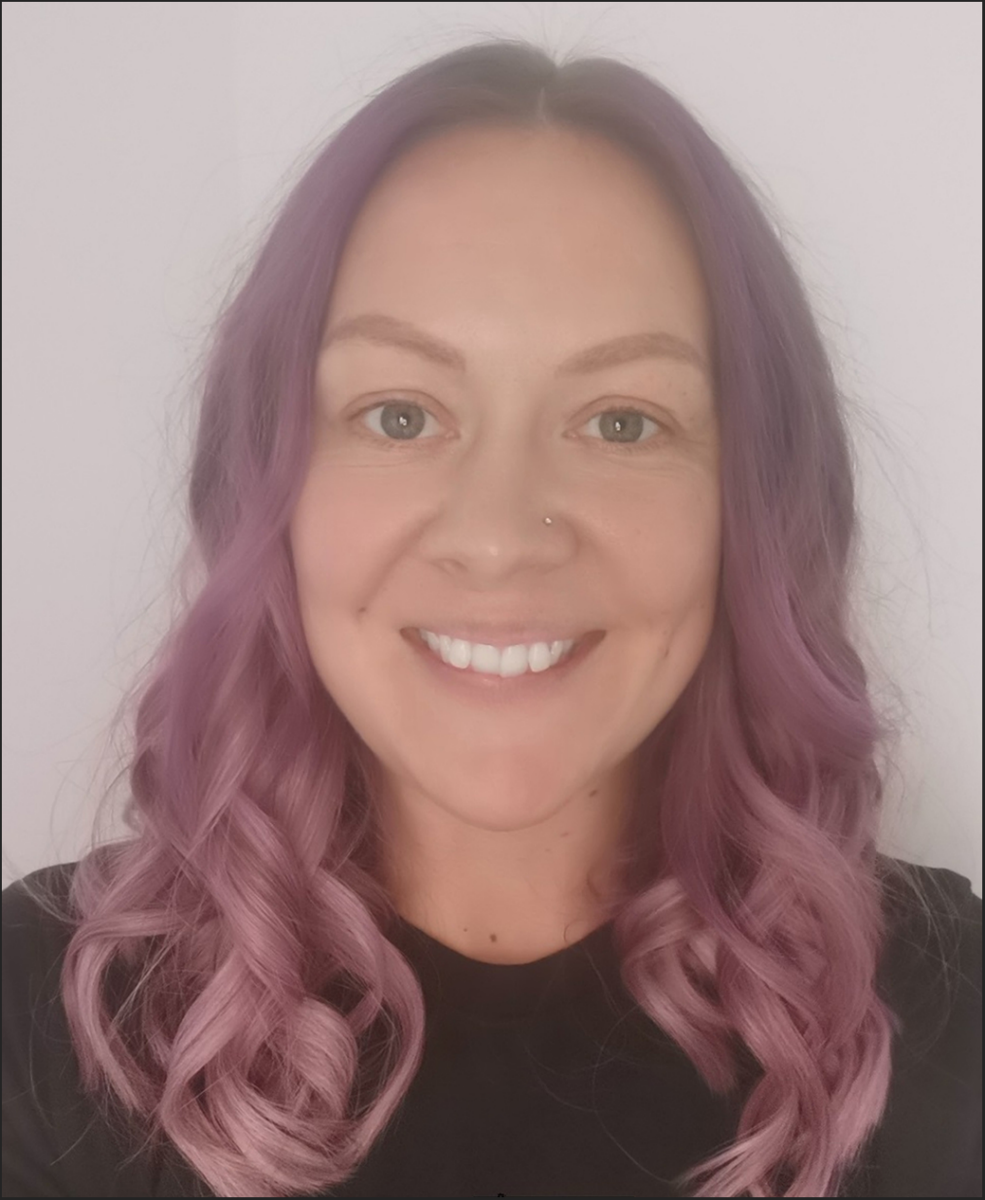


**Katie Lloyd**


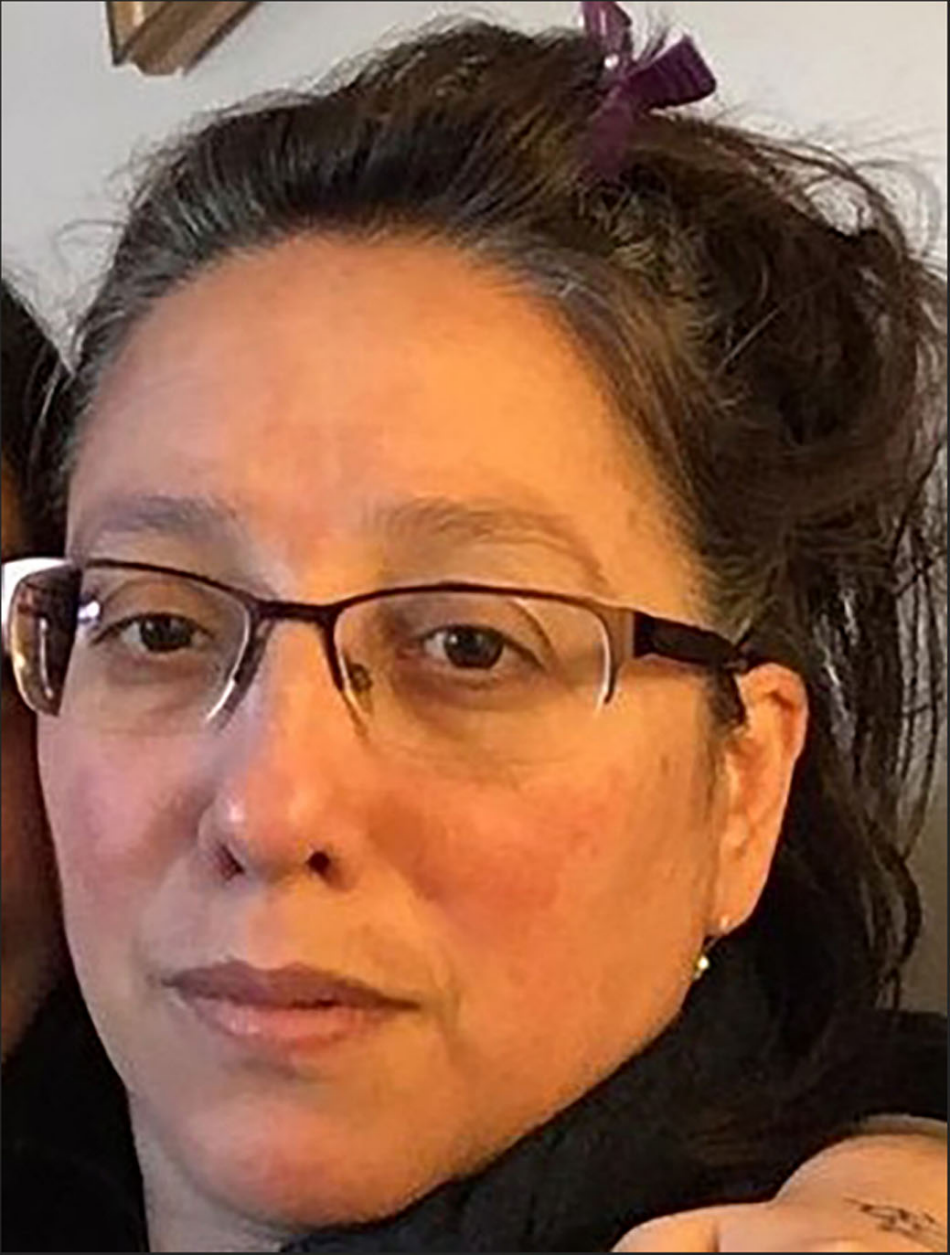


**Stamatia Papoutsopoulou**

**How would you explain the main findings of your paper to non-scientific family and friends?**

KL: We wanted to identify drugs that are already safe and well tolerated for one condition, which might also be used to treat inflammatory bowel disease (IBD). We used computerized analysis of the chemical structure of the drugs to predict whether they might work in IBD. For the most likely candidate drugs, we confirmed their effects using laboratory tests. One of the most exciting aspects of the study was the use of ‘mini-gut’ cultures grown in the laboratory from patients’ tissue samples to test the drugs.

SP: IBD is a very unpredictable medical condition with complex aggravating factors, often of unknown cause. The aim of this study was the identification and initial validation of drugs that can be further tested for IBD patient treatment. For this purpose, computational methodologies and scientific experiments were performed, mainly using cells isolated and cultured in the lab. Our results showed that the antibiotic clarithromycin can inhibit one of the major inflammatory pathways in the immune system and has the potential to be tested and used for further investigation.

**What are the potential implications of these results for your field of research?**

KL: The availability of new drugs and biological products often means new treatment options for patients. A deep understanding of the science used to create new products, testing and manufacturing procedures, and the disease/conditions that are being treated is constantly needed to bring new therapies to market. However, discovering a completely new compound is a very expensive and time-consuming process. The repurposing of already existing drugs has a huge potential to deliver new treatments to patients with incurable diseases such as IBD, quickly and cheaply. Previously developed drugs have a history of use in humans with a known safety profile and side effects and, therefore, can reduce preclinical testing and early-stage clinical trials.

SP: The drug discovery methodology we used identified the antibiotic clarithromycin as the highest-ranked drug against the transcription factor NF-κB pathway, and we further showed that it can inhibit its nuclear translocation and therefore its transcriptional activity. Clarithromycin has been an approved antibiotic for more than 20 years, and there are other new-generation antibiotics in the macrolide family. Therefore, there is a high potential for testing those drugs for their anti-inflammatory function in IBD and, possibly, in a cell-specific way, e.g. targeting cells of the immune system that are major players in the pathogenesis of the disease.

**What are the main advantages and drawbacks of the model system you have used as it relates to the disease you are investigating?**

KL: IBD is an incurable, life-long disease that is typically diagnosed in patients between the ages of 15 and 35. Due to its chronic nature, relapse is extremely common. Many patients develop resistance to drug treatments, with a significant proportion of patients requiring surgery at some point in their lifetime, so demand for new treatments is extremely high. Repurposing approved drugs would increase the potential for new therapies, particularly for non-responders who may potentially avoid surgery. However, these treatments still aim to treat inflammation that triggers symptoms as the cause of IBD is still largely unknown. Further research is required for preventative treatments in those who are at high risk of developing IBD, before the relapse/remission cycle of inflammation begins.

SP: The use of cells harvested from patients to use in live-cell experiments in the laboratory can be extremely helpful in working with specific pathways, answering specific questions and avoiding interference from external factors that can affect the outcome. The methods we have used make our experiments more relevant than ever before. Nevertheless, inflammatory diseases are multifactorial highly complex; eventually they have to be studied in patients as well as laboratory models.

**What has surprised you the most while conducting your research?**

KL: Even after studying many drug compounds through my degrees and research career, I was surprised that a number of heavily researched and commonly used drugs were predicted to affect IBD by our mathematical modelling. As the NF-κB pathway is known to be activated during chronic inflammation in IBD patients, I was expecting that anti-inflammatory drugs would be predicted by our model. We did find a number of anti-inflammatories, as expected, but it was much more exciting that drugs such as clarithromycin, a common antibiotic, and digoxin, a drug used to control heart rate, were also predicted to affect these debilitating conditions!

SP: Performing experiments in different cell types, while trying to test a hypothesis can be tedious and disappointing. In this study, I found it amazing how consistently clarithromycin could consistently inhibit the specific NF-κB pathway in macrophages under different experimental conditions *in vitro*. That included a variety of triggers to activate NF-κB signalling, different cell types and different techniques to measure NF-κB activation. These results were hugely consistent and continuously supported our original hypothesis.

**Figure:**
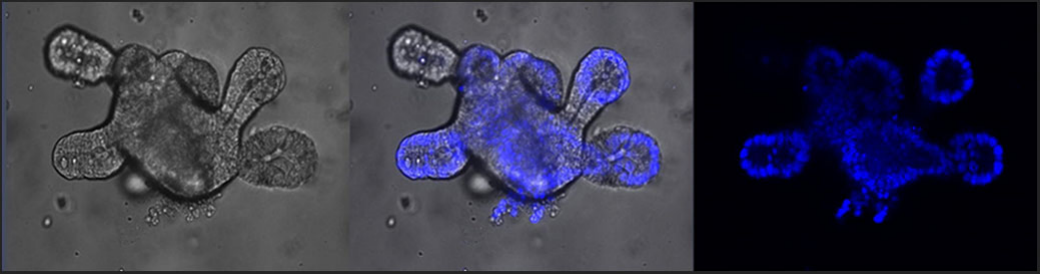
**Confocal imaging of budding small intestinal organoids showing fluorescently labelled DNA for nuclear visualisation.**

“Over the next 10 years, UK academics and universities are going to be challenged to develop new approaches that will enable our research to flourish.”

**Describe what you think is the most significant challenge impacting your research at this time and how will this be addressed over the next 10 years?**

KL: This project was funded by an EC FP7 grant awarded to a UK University. The UK's decision to leave the EU has meant that a follow-up study could not be funded through the same route and has coincided with several of the key researchers in our collaboration leaving UK universities. This has combined with the COVID-19 pandemic, to make research funding particularly difficult to obtain. Over the next 10 years, UK academics and universities are going to be challenged to develop new approaches that will enable our research to flourish.

SP: The IBDs are a chronic inflammatory condition that involves functional dysregulation of various cell types, including the immune system and the intestinal epithelium. The pathogenesis of IBD is influenced by each individual's immune system, their microbiome, environmental factors and genetic predisposition. This has been and will be the major challenge in delineating how these factors can influence the disease phenotype, behaviour and response to therapy. Cell-specific experimental approaches, such as sequencing, on large human cohorts will help to increase our understanding of the pathogenesis of the disease and promote personalised medicine.

**What changes do you think could improve the professional lives of early-career scientists?**

KL: A greater availability of funding that does not require a permanent academic position for application would allow more early-career researchers to gain experience with applying for and securing external grants to further their own independent research ideas.

SP: Early-career scientists will gain experience by being actively involved in various aspects of a scientific project, such as cost and expenses management, public engagement and interactions with stakeholders.

**What's next for you?**

KL: I have recently accepted a permanent academic position with the University of Chester, with aims to continue my research in gastrointestinal inflammation by developing my own research group at Chester Medical School and collaborations with a network of experts that I have obtained throughout my research career.

SP: I have recently accepted a permanent academic position at the University of Thessaly in Greece, where I would like to continue my research in the field of the NF-κB pathway and inflammation.
